# CoGemiR: A comparative genomics microRNA database

**DOI:** 10.1186/1471-2164-9-457

**Published:** 2008-10-06

**Authors:** Vincenza Maselli, Diego Di Bernardo, Sandro Banfi

**Affiliations:** 1 TIGEM Telethon Institute of Genetics and Medicine, Via Pietro Castellino 111, 80131 Naples Italy; 2 SEMM European School of Molecular Medicine, Naples, Italy; 3 Dipartimento Informatica e Sistemistica, Universita' degli Studi di Napoli "Federico II", Naples, Italy

## Abstract

**Background:**

MicroRNAs are small highly conserved non-coding RNAs which play an important role in regulating gene expression by binding the 3'UTR of target mRNAs. The majority of microRNAs are localized within other transcriptional units (host genes) and are co-expressed with them, which strongly suggests that microRNAs and corresponding host genes use the same promoter and other expression control elements. The remaining fraction of microRNAs is intergenic and is endowed with an independent regulatory region. A number of databases have already been developed to collect information about microRNAs but none of them allow an easy exploration of microRNA genomic organization across evolution.

**Results:**

CoGemiR is a publicly available microRNA-centered database whose aim is to offer an overview of the genomic organization of microRNAs and of its extent of conservation during evolution in different metazoan species. The database collects information on genomic location, conservation and expression data of both known and newly predicted microRNAs and displays the data by privileging a comparative point of view. The database also includes a microRNA prediction pipeline to annotate microRNAs in recently sequenced genomes. This information is easily accessible via web through a user-friendly query page. The CoGemiR database is available at

**Conclusion:**

The knowledge of the genomic organization of microRNAs can provide useful information to understand their biology. In order to have a comparative genomics overview of microRNAs genomic organization, we developed CoGemiR. To achieve this goal, we both collected and integrated data from pre-existing databases and generated new ones, such as the identification in several species of a number of previously unannotated microRNAs. For a more effective use of this data, we developed a user-friendly web interface that simply shows how a microRNA genomic context is related in different species.

## Background

MicroRNAs are a family of endogenous small non-coding RNAs with a length of approximately 21–25 nucleotides, which are usually highly conserved and predicted to regulate a large number of transcripts [1]. In the nucleus, microRNA genes are transcribed into primary transcripts (pri-microRNA) and then cleaved into shorter precursor transcripts (pre-microRNA) by the enzyme Drosha and finally exported to the cytoplasm where they are cleaved and processed by Dicer to generate mature microRNAs [2].

Concerning their genomic organization, microRNAs can be defined as a) intragenic, i.e., located within other transcriptional units (host genes), more frequently in their intronic regions and more rarely within exonic regions or b) intergenic. There is evidence that intragenic microRNAs are co-expressed with the mRNAs of their host genes [3] and therefore share with them the expression regulation control. We believe that this observation can be exploited to shed further light on the mechanisms underlying the control of microRNA expression.

As a first step towards this goal, we decided to carry out a genomic comparative analysis of microRNAs in order to gain more insight into their biology and evolution. A number of databases have already been developed to collect information about microRNA genomics, sequences and target genes [4-7]. In particular, some of them allow the user to obtain some information about the genomic organization of microRNAs, including information on the relative position with respect to other transcripts and, in the case of intragenic microRNAs, on the features of the corresponding host genes, such as miRBase, miRNAMap, miRGen and Argonaute (Table 1). However, none of them provides all of this information simultaneously and in the context of an evolutionary perspective. For this reason, we decided to develop a new microRNA-centred database with a particular focus on the genomic organization of microRNAs and of its evolutionary conservation in metazoan species.

**Table 1:** microRNA databases: differences and similarities

Feature	miRBase	miRNAMap	miRGen	Argonaute	CoGemiR
Mature sequence	+	+	-	+	+
Description	+	+	-	+	+
Genome coordinates	+	+	+	+	+
Overlapping regions	+	+	+	+	+
List of clustered MicroRNA	+	-	+	-	+
Surrounding genomic region	+	-	-	-	+
Family	+	+	-	+	+
Predicted MicroRNAs	-	+	-	-	+
List of ortholog microRNA	+	+	-	+	+
Comparative microRNA overview ^(1)^	-	-	-	-	+
Comparative location overview within species ^(2)^	-	-	+	-	+
Comparative location overview for any species ^(3)^	-	-	+	-	+
Annotation for many Metazoan	+ *	+	+	- **	+
Tissue specificity	-	+	-	-	+
Expression data	-	+	-	+	+
Link to target prediction	+	+	+	-	+
External links	+	-	-	-	+
Target section	+	-	+	-	-
List of targets	-	+	-	+	-
Stem-loop structure	+	+	-	-	+ ***
Multiple microRNAs queries ^(4)^	-	-	-	-	+

(1) The possibility to see the same microRNA in all species

(2) The possibility to see the same localization (e.g., intronic) within a species

(3) The possibility to see the same localization (e.g., intronic) in all species

(4) The possibility to submit a list of microRNAs as query

* Plus plants

** only Human, Mouse and Rat

*** only for predictions

## Results and discussion

### Construction and contents

Sequences of the annotated microRNAs were downloaded from miRBase [8,4], release 10.1, December 2007. The genome sequences, and corresponding annotation data, for all the species analyzed were extracted from the EnsEMBL database (release 48, December 2007) [9]. For each microRNA, we identified the putative host gene by querying EnsEMBL using the appropriate Perl object or running a BLAST analysis. For each intragenic microRNA, we provide information about: -the relative localization with respect to all the annotated transcripts of the overlapping gene; -the extent of conservation of the genomic location of microRNAs across evolution; -the gene expression data for the host gene, extracted from the SymAtlas database (available only for Human and Mouse) [10].

The database includes information on predicted microRNAs, which are either predicted by EnsEMBL, or by a CoGemiR pipeline based on sequence similarity procedure integrated by secondary structure analysis (Figure S1 Additional file 1). The latter procedure is similar to that implemented by the web tool miRNAminer [11] and aims at identifying microRNAs that are not yet annotated in recently sequenced genomes (see Figure S1 Additional file 1). In comparison to miRNAminer, we extended this analysis to a higher number of species. As a result, we were able to predict the presence of 188 putative microRNAs in 8 species, which are not currently annotated neither in miRBase nor in EnsEMBL (Figure S1 Additional file 1 and Table S1 Additional file 2).

**Figure 1:**
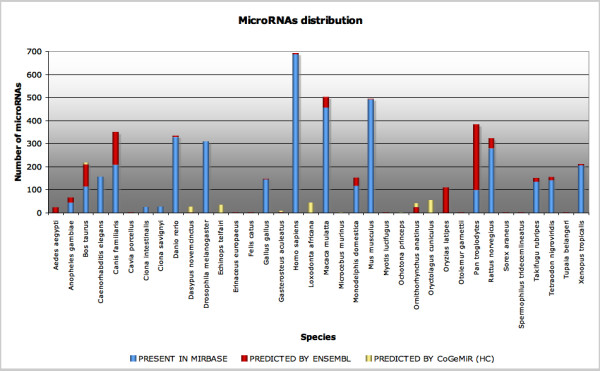
**MicroRNAs distribution across analyzed species**. The chart reports the distribution of microRNAs in different species. On the Y-axis the absolute number of microRNAs is reported, on the X-axis the name of the species. In yellow the CoGemiR high confidence (HC) prediction, in red the EnsEMBL prediction and in blue the microRNA present in miRBase.

Overall, CoGemiR contains 5064 records – 3970 of which are annotated in miRBase, 906 are predicted by EnsEMBL and 188 are predicted by CoGemiR – distributed across 36 species, as shown in Figure 1. Furthermore, we provide direct links to miRBase and EnsEMBL, direct link to taxonomy information for all the species analyzed and direct links, whenever possible, to other relevant database, including web sites for target prediction.

### Utility

The information contained in CoGemiR are both collected from pre-existing databases and newly generated via a processing pipeline we implemented in order to determine microRNA genomic organization and conservation across a number of metazoan species.

The database web interface allows verifying whether a given microRNA is reported to be intragenic or intergenic in one or more species. For intragenic microRNAs, it is possible to verify whether or not a particular microRNA is located within the same gene in different species as well as to establish whether its position within the host gene (e.g., exonic, intronic, etc.) is also conserved. In addition, for most of intragenic microRNAs, we provide both qualitative and quantitative expression information pertaining to the corresponding overlapping genes. A specific filter allows the user to restrict the search only to already annotated microRNAs or to those predicted by EnsEMBL and/or CoGemiR (see next section for further details).

### User Interface

**Figure 2:**
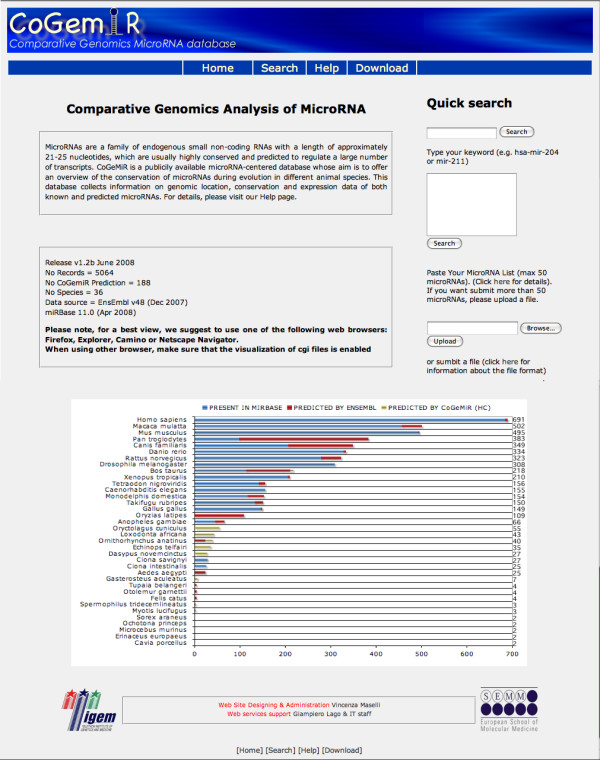
**Main query page**. The CoGemiR home page allows the user to make a quick search (more details in the text).

**Figure 3:**
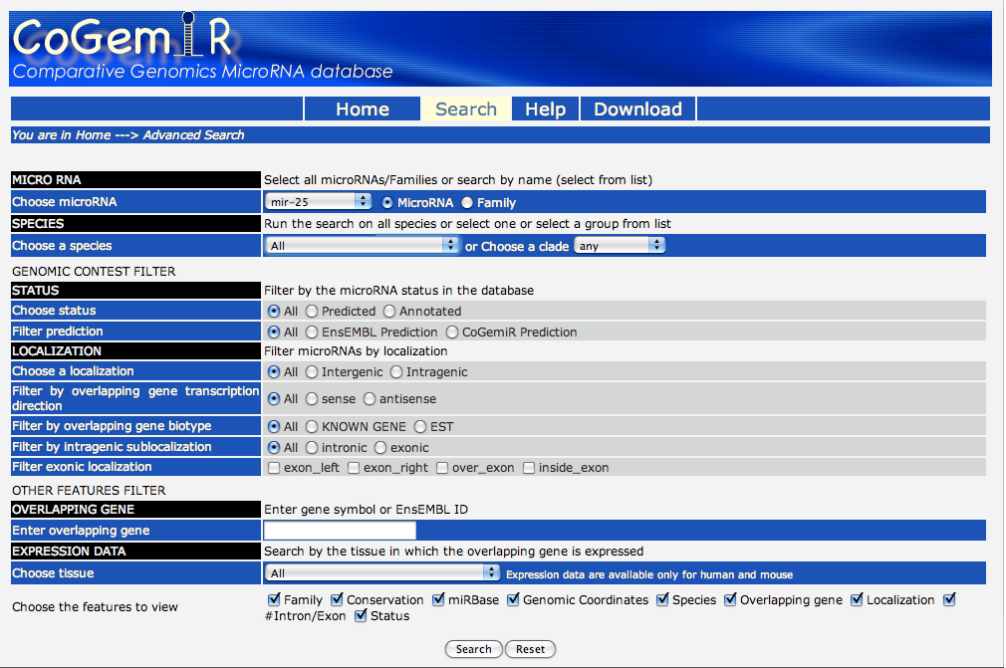
**Search page**. In the CoGemiR search page a user-friendly form guides the user in performing the queries.

The main page briefly summarizes CoGemiR aims, provides some statistics and allows the user to submit a quick search (Figure 2). There are tree ways to perform a quick search, i.e., (i) by typing a keyword (e.g., mir-124), (ii) by pasting a list of microRNAs (max 50) or (iii) by uploading a file. The query mask allows the user to perform more complex queries. In the advanced search, all the possible search options are available. In the main search page, the query becomes more and more specific in a step-by-step process, depending on the user's choice. It is possible to retrieve: (1) a specific microRNA in a selected species; (2) the list of microRNAs present in a given species or in a set of species (e.g., all human microRNAs or microRNAs present in all Primates); (3) the list of the species in which a selected microRNA or microRNA family is present. In the latter two cases, the search can also be further restricted by 1) microRNA 'status', i.e., to allow the user to retrieve either predicted or annotated microRNAs only; by 2) 'localization', i.e., to allow retrieval of all the microRNAs with a specific localization (intergenic or intragenic and within the latter category exonic, intronic, etc.); and by 3) expression data, i.e., to allow retrieval of all the microRNAs with an overlapping gene expressed in a specific tissue (option available only for human and mouse). Additional and more complex subqueries are possible by combining status and localization filters (Figure 3). Parts of the form appear only if a filter is selected (e.g., the specific localization filter appear only if intragenic microRNAs are selected). Using wildcards (a question mark immediately after the microRNA name), it is possible to perform less stringent queries, e.g., by entering the query "mir-124?" will yield as results both "mir-124" and "mir-124a" entries.

**Figure 4:**
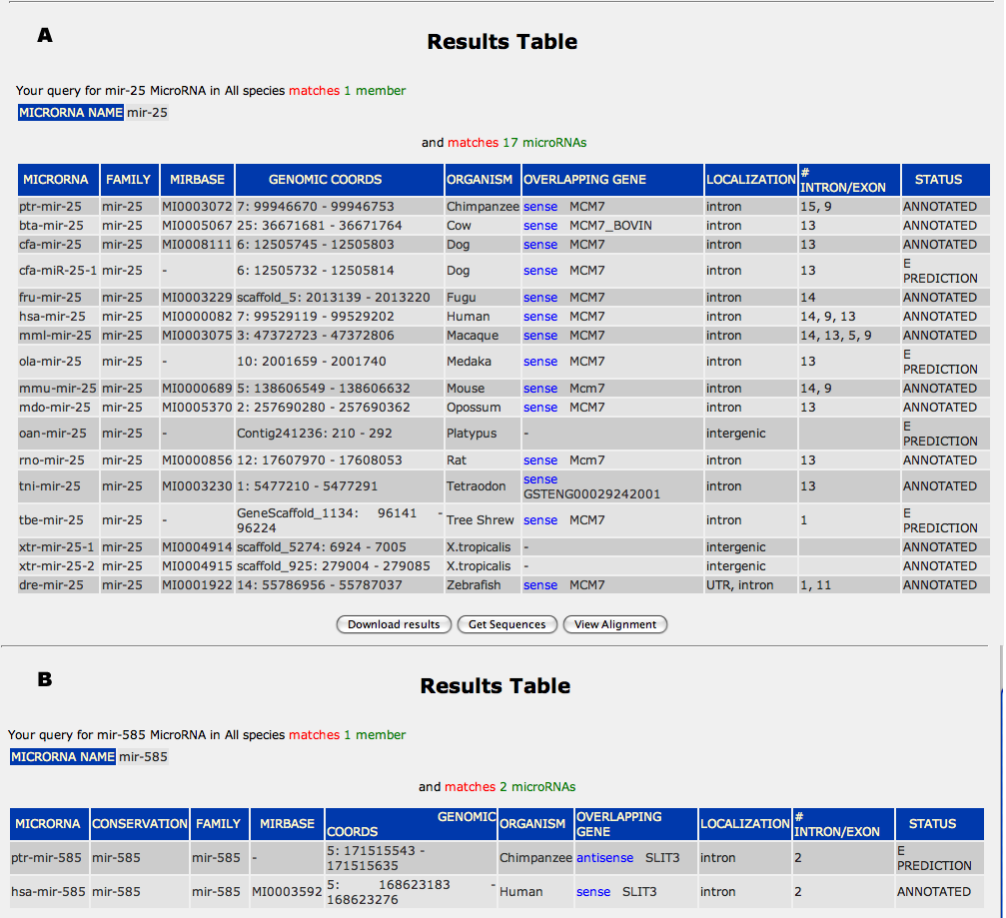
**The Result page**. The CoGemiR Results table summarizes the main information about the queried microRNAs. Panels A and B show the results obtained after querying the database, respectively, with the tems "miR-25" and "mir-585" in all species (more details are provided in the text). Please note that the gene GSTENG00029242001 listed in panel A is the tetraodon homolog of the Mcm7 gene, as assessed by EnsEMBL analysis.

The "Results" page contains a summary of the main information about the queried microRNAs (Figure 4). The user can choose the columns to be visualized. The first column ("MicroRNA") reports the name of the microRNA linked to the "extra feature" page (see below), the "conservation" column, which only appears when a single species is selected in the query, provides a link to the list of species in which the microRNA is present, the "family" column provide a link to the list of the microRNAs belonging to the same family in all species, the "miRBase" column provides a direct link to miRBase whereas the "genomic coordinates" column and the "overlapping gene" column, which contain the name of the gene overlapping the microRNA, provide links to EnsEMBL (release 48). The last three columns indicate the microRNA localization, the rank of the intron/exon of the gene in which intragenic microRNAs are embedded and the status of the microRNA, i.e., annotated or predicted. For the CoGemiR predicted microRNAs, the "status" column provides a link to the secondary structure prediction data. As shown in Figure 3, the "Results" page allows to obtain an evolutionary overview of microRNA genomic features. For example, it is possible to simultaneously assess the genomic localization of a given microRNA in all the analyzed species, as shown in Figure 4.

Finally, below the "results page" table, there are links for downloading the sequences of the retrieved microRNAs in FASTA format and visualizing them in a multiple sequence alignment format (generated by ClustalW), which allows the recognition of the nucleotides that have diverged across evolution in different species. A phylogenetic tree in Phylip format is also provided.

**Figure 5:**
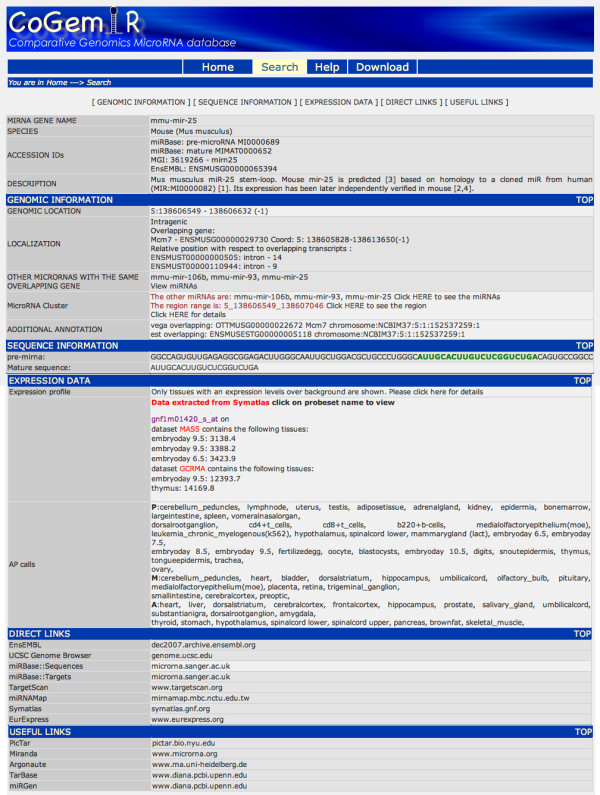
**The extra feature page**. The CoGemiR Extra feature page shows a list of other additional information about the queried microRNA.

The "Extra features" page, accessible via the "microRNA" link in the result page collects additional information (Figure 5), such as the genomic coordinates of both the microRNA and the overlapping gene, additional information on the genomic region, including the presence of nearby microRNAs that may be part of a microRNA cluster and/or additional overlapping transcribed sequences (e.g., ESTs), the sequences of both the precursor and mature forms of microRNAs, microarray expression data of the overlapping gene (extracted from SymAtlas [12]) and links to other external databases. Please note also that all query results can be downloaded by the user.

### Comparison and improvement on similar existent database

The main difference between CoGemiR and already available resources [4-7], is represented by the evolutionary perspective of data presentation. Other already available microRNA databases already contain information on microRNA genomic data and evolutionary conservation but none of them allows the simultaneous and systematic retrieval of these data for each microRNA. These features render CoGemiR a valuable resource to gain insight into the evolution of the genomic organization of microRNAs. For instance, the use of CoGemiR allows to assess that the genomic organization of miR-25 is likely to be highly conserved across evolution, Figure 4A shows that this microRNA is intragenic and is localized within the same host gene, *MCM7*, in all species analyzed, with the possible exception of Xenopus tropicalis. On the other hand, it is possible to identify an intriguing difference in the genomic organization of miR-585, a microRNA so far identified only in human and in chimp. This microRNA is localized within the *SLIT3 *gene in both species but in different orientations, being transcribed in opposite orientation with respect to *SLIT3 *in chimp and in the same orientation in human (Figure 4B). Since it was suggested that an intragenic microRNA and the corresponding hosting transcriptional unit share both expression profiles and expression control elements when they are transcribed in the same direction [3], this difference may imply a significant difference in the regulation of expression of miR-585 between human and chimp. Obviously, the verification of this hypothesis requires further investigation.

Moreover, similar to miRNAMiner [11], CoGemiR allows to perform an automatic annotation of newly sequenced genomes for microRNAs. However, compared to miRNAMiner, we extended the latter analysis to a higher number of species.

Table 1 summarizes the main features of CoGemiR in comparison with other available resources.

## Conclusion

MicroRNAs are small molecules, which play a crucial role in gene regulation. Several functional genomics efforts are currently in progress to help deciphering their function in basic biological processes. We believe that a detailed knowledge of the relationship between microRNA and their genomic environment provides extremely valuable information towards a better understanding of their functional role. Towards this goal, we developed CoGemiR, a database that allows an effective retrieval of microRNA genomic data and of their extent of conservation across evolution.

Compared to other preexisting databases, CoGemiR is more "user-friendly" and contains significant elements of novelty (see Table 1). We plan to improve our tool including the possibility of searching the database by sequence.

## Methods

The dataset is stored in a relational form using MySQL 4.1.14, whereas all the necessary scripts to store and query the database were written in Perl v5.8.8 (built for i686-linux) object-oriented scripting language, making use of two non-standard libraries, EnsEMBL Perl, [13] and BioPerl [14]. The web interface was written using Perl CGI library.

The CoGemiR database is available at . For a best view, we suggest to use one of the following web browsers: Firefox, Explorer, Safari, Camino or Netscape Navigator. When using other browser, make sure that the visualization of cgi files is enabled.

## Authors' contributions

VM designed and constructed the database and the website, was involved in data curation, and drafted the manuscript. DdB participated to the design of the project, organization of the web site and helped to draft the manuscript. SB conceived and coordinated the study and wrote the final version of the manuscript. All authors read and approved the final manuscript.

## Supplementary Material

Additional file 1 **Flowchart of the analysis used to identify unannotated microRNAs in the genomes analyzed.** The additional file 2 contains a flowchart of the microRNAs prediction pipeline. Click here for file

Additional file 2 **List of CoGemiR predicted microRNAs.** The additional file 1 contains a table in which the list of CoGemiR predicted microRNAs is reported. Click here for file
