# Supplementary material for: CoGemiR: A comparative genomics microRNA database
Source: BMC Genomics. 2008 Oct 6;9:457. doi: 10.1186/1471-2164-9-457 (PMC2567348; doi:10.1186/1471-2164-9-457)
Supplement: Additional file 1 — Flowchart of the analysis used to identify unannotated microRNAs in the genomes analyzed. The additional file 2 contains a flowchart of the microRNAs prediction pipeline. [file 1471-2164-9-457-S1.doc]

##
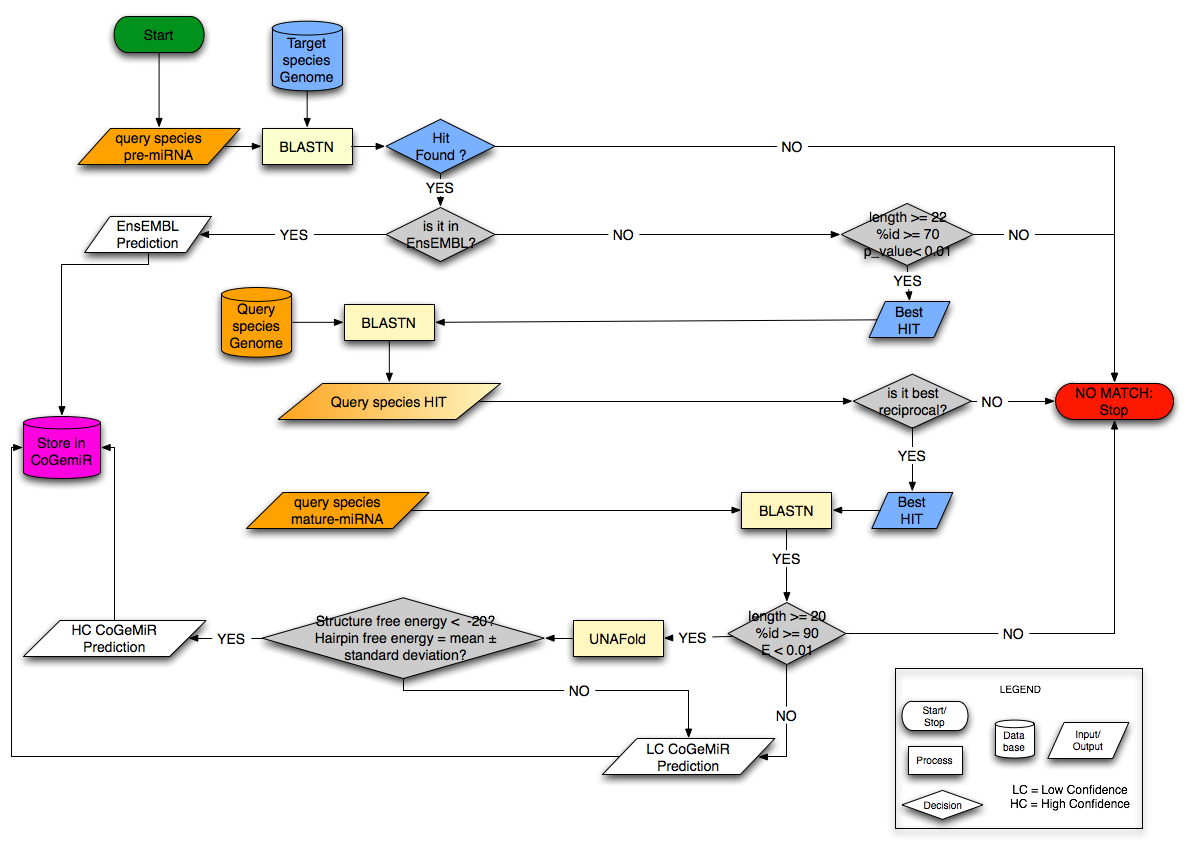
Figure S1 Flowchart of the analysis used to identify unannotated microRNAs in the genomes analyzed.

The pipeline begins by selecting the precursor sequences of all microRNAs from a reference species (Human, Mouse, Rat, Chicken, X. tropicalis, Zebrafish, Fugu, Drosophila and C. elegans), which are not annotated in at least one of the species analyzed, as assessed from the analysis of the miRBase and EnsEMBL databases. We then ran BLASTN against the genomic sequence of the “microRNA-missing” species. In case of BLAST sequence hits satisfying the thresholds of: length > 70 nt, percentage id (%)  > 70% and Evalue (E) < 0.01, we ran another BLASTN using the detected hit as query against the human genome sequence (reciprocal Blast). If this analysis revealed a best hit corresponding to the starting microRNA precursor sequence, we ran a third BLASTN analysis to compare the hit sequence with the mature sequence of the starting microRNA. If the resulting percentage identity, Evalue and the length passed the thresholds (respectively, l > 20 nt, (%)  > 90% and (E) < 0.01), we performed a secondary structure prediction of the new microRNA by using the UNAFold software [1]. If at least one predicted structure has a free energy value lower than -20 and has a hairpin with a free energy value which is near the average free energy value of the hairpin of the homologous, already annotated microRNAa (plus or minus the standard deviation), the microRNA is labeled as a High Confidence Prediction (HC) otherwise it is labeled as Low Confidence Prediction (LC) Both are stored into the database but only the HC will be considered in the further analysis. We tested the specificity (98%) and the sensitivity (76%) of our prediction procedure by using the entire data set of human microRNAs against the mouse genome sequence.

1. Markham NR, Zuker M**: UNAFold: software for nucleic acid folding and hybridizati**on*. Methods Mol Bio*l 2008**, 4**53:3-31.
