# Supplementary material for: CoGemiR: A comparative genomics microRNA database
Source: BMC Genomics. 2008 Oct 6;9:457. doi: 10.1186/1471-2164-9-457 (PMC2567348; doi:10.1186/1471-2164-9-457)
Supplement: Additional file 2 — List of CoGemiR predicted microRNAs. The additional file 1 contains a table in which the list of CoGemiR predicted microRNAs is reported. [file 1471-2164-9-457-S2.doc]

Table S1

| Species | List of CoGemiR predicted microRNAs |
| --- | --- |
| Echinops telfairi | ete-miR-138-1, ete-mir-142, ete-mir-196b, ete-mir-23b, ete-mir-223, ete-mir-31, ete-mir-208, ete-mir-33a, ete-mir-671, ete-mir-107, ete-mir-34a, ete-mir-154, ete-mir-132, ete-mir-136, ete-mir-383, ete-mir-181c, ete-mir-186, ete-mir-889, ete-mir-192, ete-mir-200a, ete-mir-361, ete-mir-34c, ete-mir-184, ete-mir-27b, ete-mir-137, ete-mir-205, ete-mir-504, ete-mir-15b, ete-mir-145, ete-mir-340, ete-mir-381, ete-mir-592, ete-mir-215, ete-mir-380 , ete-mir-495 |
| Loxodonta africana | laf-mir-378, laf-mir-195, laf-mir-708, laf-mir-92b, laf-mir-31, laf-mir-208, laf-mir-452, laf-mir-30a, laf-mir-155, laf-mir-671, laf-mir-363, laf-mir-652, laf-mir-433, laf-mir-154, laf-mir-132, laf-mir-140, laf-mir-369, laf-mir-181c, laf-mir-186, laf-mir-889, laf-mir-361, laf-mir-22, laf-mir-184, laf-mir-451, laf-mir-615, laf-mir-211, laf-mir-137, laf-mir-374b, laf-mir-205, laf-mir-490, laf-mir-505, laf-mir-182, laf-mir-21, laf-mir-592, laf-mir-181d, laf-mir-532, laf-mir-582, laf-mir-497, laf-mir-376c, laf-mir-299, laf-mir-377, laf-mir-106b, laf-let-7d |
| Microcebus murinus | mmr-mir-10b, mmr-mir-190 |
| Dasypus novemcinctus | dno-miR-138-1, dno-mir-375, dno-mir-142, dno-mir-127, dno-mir-551b, dno-mir-454 ,dno-mir-30b, dno-mir-31, dno-mir-671, dno-mir-154, dno-mir-140, dno-mir-186, dno-mir-30e, dno-mir-192, dno-mir-29a, dno-mir-451, dno-mir-760, dno-mir-205, dno-mir-182, dno-mir-145, dno-mir-128a, dno-mir-362, dno-mir-532, dno-mir-330, dno-mir-495 |
| Gasterosteus aculeatus | gac-miR-9-2, gac-mir-23b, gac-mir-27b, gac-mir-137, gac-mir-455 |
| Oryctolagus cuniculus | ocu-miR-138-1, ocu-miR-124-1, ocu-miR-153-1, ocu-miR-125b-1, ocu-miR-194-1, ocu-mir-375, ocu-mir-26b, ocu-mir-142, ocu-mir-551b, ocu-mir-502, ocu-mir-188, ocu-mir-195, ocu-mir-324, ocu-mir-208, ocu-mir-30a, ocu-mir-217, ocu-mir-671, ocu-mir-363, ocu-mir-107, ocu-mir-34a, ocu-mir-154, ocu-mir-140, ocu-mir-186, ocu-mir-346, ocu-mir-889, ocu-mir-30e , ocu-mir-361, ocu-mir-34c, ocu-mir-96, ocu-mir-496, ocu-mir-206, ocu-mir-137, ocu-mir-374b, ocu-mir-151, ocu-mir-760, ocu-mir-490, ocu-mir-99a |
| Ornithorhynchus anatinus | oan-miR-138-1, oan-miR-124-1, oan-mir-142, oan-mir-363, oan-mir-302b, oan-mir-367, oan-mir-490, oan-mir-221, oan-mir-21, oan-mir-302c, oan-mir-215 |
| Ochotona princeps | opr-mir-203, opr-mir-451 |
